# Comparison of COVID-19 Inactivated Virus Vaccine Immunogenicity Between Healthy Individuals and Patients on Hemodialysis: A Single-Center Study From Pakistan

**DOI:** 10.7759/cureus.24153

**Published:** 2022-04-15

**Authors:** Sapna Bai, Murtaza Dhrolia, Hina Qureshi, Ruqaya Qureshi, Kiran Nasir, Aasim Ahmad

**Affiliations:** 1 Nephrology, Jinnah Postgraduate Medical Centre, Karachi, PAK; 2 Nephrology, The Kidney Centre Postgraduate Training Institute, Karachi, PAK; 3 Hematology, The Kidney Centre Postgraduate Training Institute, Karachi, PAK

**Keywords:** maintenance hemodialysis, hemodialysis, covid antibody titer, covid-19 vaccine, chronic kidney disease

## Abstract

Introduction

This study compares the immune response after coronavirus disease 2019 (COVID-19) inactivated virus vaccine between healthy individuals (HI) and patients on hemodialysis (HD).

Methods

In this cross-sectional, comparative study, the presence or absence of immunoglobulin G (IgG) anti-S antibody and IgG anti-S antibody titer was compared between HI, and patients on HD after two doses of COVID-19 vaccine.

Results

A total of 81 participants, 50 (61.7%) HD patients and 31 (38.3%) HI, were studied. The mean age was 52.9±12 in HD patients and 42±12.4 in HI. Vaccination responder rates were 80.6% in HI and 72% in HD patients after the first dose (p=0.38) and 93.5% in HI and 94% in HD at the third week of the second dose of the vaccine (p=0.93). The mean IgG antibody titer was 156.3±113.8 in HI and 143.4 ± 117.8 in HD patients (p=0.538) after the first dose and 186.7 ± 97.9 in HI and 180.6 ± 105.8 in HD patients (p=0.552) at three weeks of the second dose. No statistically significant difference was found in antibody titer with respect to gender, age, vaccine (BBIBP-CorV or Conovac), and hypertension. Diabetic HD patients had a lower antibody titer than non-diabetic HD patients (p=0.03) while participants who had a history of COVID-19 infection had a higher IgG titer (p = 0.001). The levels of IgG titer in the same patient increased, corresponding to the doses of vaccine (p <0.001). No HD patient developed COVID-19 infection till the third week of vaccination.

Conclusion

This study demonstrates a similar humoral response after COVID-19 inactivated virus vaccination in HD patients and HI. The response was lower among diabetic patients on HD and better in those with previous COVID-19 infection.

## Introduction

The sudden emergence of the coronavirus disease 2019 (COVID-19) pandemic raised serious health threats globally, with devastating outcomes for hemodialysis (HD) patients worldwide, with mortality ranging from 21% to 32.8% in different studies [1-4], much higher than that for the general population [2-3]. The large population size of HD patients [5-6], the compromised immune system [7], along with the multiple comorbidities, such as diabetes mellitus (DM), hypertension (HTN), and ischemic heart disease (IHD), make them more susceptible not only to COVID-19 infection but also to severe illness. HD patients need special consideration in this respect.

Generally, non-pharmacological interventions to reduce the risk of COVID infection, such as a facial mask mandate, regular severe acute respiratory syndrome coronavirus 2 (SARS-CoV-2) testing, and isolation measures for patients with COVID-19, are efficacious. However, maintenance hemodialysis (MHD) patients at hemodialysis centers are in contact with healthcare workers (HCWs) and other patients in a relatively confined environment for a considerably long time, making them more susceptible. Therefore, it is more challenging for health care authorities to prevent the spread and manage infectious diseases in these patients than in the general population [8-9]. Effective COVID-19 vaccination would be of great clinical importance in such patients. The licensing of novel vaccines against SARS-CoV-2 in early 2021 enabled global vaccine roll-outs that have significantly reduced mortality risks in the general population. However, most COVID-19 vaccination studies had excluded patients with chronic kidney disease (CKD) and the HD population.

CKD is associated with immunodeficiency, and vaccines such as those for hepatitis B need a double-dosing regimen for patients with CKD [10]. However, patients with CKD appear to seroconvert at a similar rate to the general population after a COVID-19 infection [11], suggesting vaccine efficacy. Therefore, data on vaccine efficacy and immunological responses from healthy cohorts have limited relevance to cohorts of CKD patients, and it remains unclear if current SARS-CoV-2 vaccination approaches are suitable for these patients.

The literature so far available regarding the response rate of COVID-19 vaccination in HD patients is limited; most studies were conducted in Western nations, and only mRNA and virus vector-based vaccines were used [12-13]. Variable response rates across these clinical trials may be explained by different vaccine types, vaccine doses, criteria for a positive response, timings of antibody detection, races, and ethnicities. To fill the gap, we aimed to evaluate the difference between antibody response achieved after COVID-19 vaccination between healthy individuals (HI), and patients on HD, after inactivated virus vaccine (Sinopharm BBIBP-CorV or Sinovac-CoronaVac) in our study.

## Materials and methods

This cross-sectional, comparative, single-center study was conducted in the Department of Nephrology at The Kidney Centre Postgraduate Training Institute (TKC-PGTI) after approval by the institutional ethical review committee. TKC-PGTI is a tertiary-level renal care facility with a dialysis unit that accommodates 66 HD patients and performs 82,500 dialysis sessions annually, following standards defined by European best-practice guidelines [14]. Nearly all long-term maintenance HD patients are dialyzed for four hours, thrice a week.

We compared the immune response between HD patients and healthy individuals in our center after COVID-19 inactivated virus vaccination (BBIBP-CorV (Sinopharm Beijing Institute of Biological Products COVID-19 vaccine) or ConoVac (Sinovac-CoronaVac COVID-19 vaccine)). All in-center on-dialysis patients and health care workers or partners, siblings, or family members of the in-center patient who received approved BBIBP-CorV or Conovac COVID-19 vaccines and volunteered to participate in the study were included using the non-probability convenience sampling technique. Written informed consent was taken. Exclusion criteria included the following: individuals under 18 years of age, history of severe adverse reaction associated with a vaccine and/or severe allergic reaction (e.g., anaphylaxis) to any component of the study intervention(s), multiorgan transplant recipients, patients on immunosuppressive therapy, pregnant or breastfeeding women, patient with active (hematological) malignancy, inherited immune deficiency, infection with human immunodeficiency virus (HIV), and bleeding diathesis or condition associated with prolonged bleeding that would that contraindicate intramuscular injection.

Data collection procedure

COVID-19 vaccination was done according to the standard of care: two doses, 21 days apart, for BBIBP-CorV and ConoVac. Blood was drawn at three different time points: baseline (before the first vaccine dose), on the 20th day (before the second vaccine dose), and at three weeks after the second dose of vaccination. The presence or absence of immunoglobulin (IgG) anti-S antibody and IgG anti-S antibody titer was compared between healthy individuals and patients on hemodialysis. Testing was performed on Cobas® e 411 by using Cobas® Elecsys Anti‐SARS‐CoV‐2 S Immunoassay (Roche Diagnostics, Basel, Switzerland) for the in vitro quantitative determination of high-affinity antibodies (including IgG) to the SARS‐CoV‐2 spike (S) protein receptor-binding domain (RBD) in human serum and plasma in a double-antigen sandwich assay. The IgG anti-S antibody detection limit was 0.4 U/mL to 250 U/ML. Results below or above these detection limits were defined as lesser than 0.4 U/mL or more than 250 U/mL. The result was interpreted as negative for anti‐SARS‐CoV‐2‐S if less than 0.8 U/mL and positive for anti‐SARS‐CoV‐2‐S if more than or equal to 0.8 U/uL.

The COVID-19 incidence will be calculated as the number/percentage of patients who develop COVID-19 infection after vaccination before the second vaccine dose and three weeks after vaccination (i.e., the second dose). Demographic, clinical, and laboratory data were collected in the form of a proforma.

Biospecimen retention

Samples without DNA were in total 3 x 10 ml of heparin blood, 15 ml ethylenediaminetetraacetic acid (EDTA) blood, and 10 mL serum will be drawn and kept until the end of the study, and no other tests will be conducted done on them.

Statistical analysis 

The data were entered and analyzed on IBM SPSS version 21 (IBM Corp., Armonk, NY). Cleaning and coding of data were done prior to analysis. Mean ± standard deviation was computed for continuous variables while the frequency with percentage was calculated for categorical variables. To observe the difference in parameters of both groups of participants, the chi-square or Fisher's exact test was applied as appropriate. The Mann-Whitney U-test was executed to find any difference in IgG antibody titer between the parameters of study participants in the case of two independent samples while the Kruskal Willis test was used in more than three groups. The Friedman test was applied for the repeated measure. Shapiro Wilk’s test checked the normality of data. The significant level was set as ≤ 0.05.

## Results

We recruited a total of 81 participants in our study, of which 50 (61.7%) were dialysis patients and 31 (38.3%) were healthy individuals. Males dominated in both groups, with 25 (80.6%) healthy individuals and 34 (68%) HD patients. The mean age of dialysis patients was 52.9±12 while the mean age of healthy individuals was 42±12.4. Patients on HD had more comorbidities compared to healthy individuals (Table 1). 

**Table 1:** Baseline characteristics of participants BBIBP-CorV = Sinopharm Beijing Institute of Biological Products COVID-19 vaccine ConoVac = Sinovac-CoronaVac COVID-19 vaccine COVID-19 = Corona Virus Disease of 2019 SD = Standard Deviation

Variables		Total		Healthy Individuals		Hemodialysis Patients	
		(n = 81)	n%	(n = 31)	n%	(n = 50)	n%
Gender (n/%)	Male	59	72.8	25	80.6	34	68
	Female	22	27.2	6	19.4	16	32
Vaccine (n/%)	BBIBP-CorV	42	51.9	21	67.7	21	42
	ConoVac	39	48.1	10	32.5	29	58
Diabetes mellitus (n/%)		21	25.9	0	0	21	44
Hypertension (n/%)		44	54.3	4	12.9	40	80
Ischemic heart disease(n/%)		7	8.6	0	0	7	14
History of COVID-19 infection (n/%)		18	22.2	8	25.8	10	20
Age (mean±SD)		48.8±13.8		42.1±12.4		52.9±13	

Vaccination responder rates were 80.6% (25/31) in healthy individuals and 72% (36/50) in HD patients after the first dose (p=0.38) and 93.5% (29/31) in healthy individuals and 94% (47/50) on HD at the third week of the second dose of the vaccine (p=0.93). Gender, age, type of vaccine (BBIBP-CorV or Conovac), and HTN did not show a statistically significant difference in vaccine response. Diabetic HD patients showed poor vaccine response than non-diabetic HD patients after the first dose of the vaccine (p = 0.005). However, the responder rate was not statistically significant at the third week of the second dose (p=0.07). The participants who had a history of COVID-19 infection previously had a better response rate than patients without this history after the first dose (p=0.006). However, this was not maintained as statistically significant at the third week of the second dose (p=0.22) (Table 2).

**Table 2:** Comparison of vaccine responders according to their baseline characteristics *Non-responder = IgG anti-S antibody titer <0.8 U/mL **Responder = IgG anti-S antibody titer ≥0.8 U/mL IgG = immunoglobulin G

Parameters		n					
		At the 20^th^ day after the 1st dose			At the 3^rd^ week of the 2^nd^ dose		
		Non-responder	Responder **	P-value	Non-responder *	Responder **	P-value
Gender	Male	14	45	0.74	3	56	0.5
	Female	6	16		2	20	
Age	≤ 35 years	4	12	0.65	0	16	0.06
	36 – 50 years	5	24		0	29	
	51 – 60 years	5	10		3	12	
	> 60 years	6	15		2	19	
Participant	Healthy volunteer	6	25	0.38	2	29	0.93
	Hemodialysis patient	14	36		3	47	
Diabetes mellitus	Yes	10	11	0.005	3	18	0.07
	No	10	50		2	58	
Hypertension	Yes	13	31	0.27	3	41	0.79
	No	7	30		2	35	
Vaccine	Sinopharm	10	32	0.85	2	40	0.58
	Sinovac	10	29		3	36	
History of COVID	Yes	0	18	0.006	0	18	0.22
	No	20	43		5	58	

When we observed the difference in response of the IgG antibody titer at different times, the mean IgG antibody titer was 156.3±113.8 in healthy individuals and 143.4±117.8 in HD patients (p=0.538) after the first dose of the vaccine and 186.7±97.9 in healthy individuals and 180.6±105.8 in HD patients (p=0.552) at three weeks of the second dose. Gender, age, type of vaccine (BBIBP-CorV or Conovac), and HTN did not significantly impact the antibody titer. DM and a positive history of COVID infection had a statistically significant impact on IgG titer. The diabetic HD patients had a low mean titer of antibody compared to non-diabetic HD patients after administration of the first dose of the vaccine (96.4±116 v/s 166.5±111, respectively). The same observation was also found at the third week of the second dose of the vaccine (p=0.03). The participants who had a history of COVID-19 infection previously had a higher mean IgG titer than patients without this history. The difference was found at all levels before and after administering vaccines (p = 0.001). We also noticed that at the third week of the second dose, all participants with a positive history of COVID-19 infection achieved maximum levels of the IgG titer (250±000) (Table 3).

**Table 3:** Comparison of the IgG antibody titer of participants according to their baseline characteristics * = statistically significant IgG = immunoglobulin G

Parameters		Mean ± STD of IgG antibody titer at three different times of administration of Vaccine					
		Baseline	P-value	Before the 2^nd^ dose	P-value	At the 3^rd^ week of the 2^nd^ dose	P-value
Gender	Male	94 ± 118.9	0.796	147.1 ± 145.6	0.835	103.3 ± 13.5	0.211
	Female	118.4 ± 120.8		151.5 ± 121.7		197.5 ± 100.4	
Age	≤ 35 years	62.4 ± 23.3	0.684	138.7 ± 118.6	0.297	188.1 ± 101	0.74
	36 – 50 years	124.1 ± 138.2		168.2 ± 112.3		194.5 ± 93.4	
	51 – 60 years	87 ± 113.9		126.3 ± 122.1		172.5 ± 115.2	
	> 60 years	107 ± 111.2		1444 ± 118.1		170.5 ± 107	
Participant	Healthy Volunteer	94.1 ± 129.8	0.439	156.3 ± 113.8	0.538	186.7 ± 97.9	0.552
	Hemodialysis patient	104.7 ± 113.3		143.4 ± 117.8		180.6 ± 105.8	
Diabetes mellitus	Yes	88.9 ± 113.9	0.376	96.4 ± 116	0.008*	143.6 ± 117.3	0.03*
	No	104.7 ± 121.6		166.5 ± 111		196.7 ± 93.7	
Hypertension	Yes	109.2 ± 111.8	0.213	156.2 ± 115.9	0.828	186.5 ± 102	0.886
	No	90.4 ± 128.2		139 ± 116.5		178.6 ± 103.9	
Vaccine	Sinopharm	89.6 ± 107.6	0.621	144.1 ± 116.9	0.861	183.4 ± 103.6	0.745
	Sinovac	112.5 ± 130.9		152.9 ± 115.9		182.4 ± 102.2	
History of COVID	Yes	174.7 ± 91.8	0.001*	223.7 ± 76.5	0.001*	250 ± 000	0.001*
	No	79.5 ± 118.2		126.8 ± 116.5		163.71 ± 108.7	

The levels of IgG titer increased in the same patient corresponding to the doses of vaccine (p <0.001). This was observed both in healthy individuals as well as patients on HD. (p=0.909) (Table 3).

We further observed the antibody response of participants according to their baseline IgG titer and discovered that antibody response was significantly greater in participants who had a baseline antibody titer of > 5 as compared to ≤ 5 (p <0.001) (Table 4).

**Table 4:** Comparison of the response of the COVID vaccine between ≤ 5 and > 5 baseline IgG antibody titer of participants IgG = immunoglobulin G

IgG antibody titers	With baseline ≤ 5 mean ± STD	With baseline > 5 mean ± STD	P-value
Before 2^nd^ vaccine dose	26.5 ± 64	232.1 ± 49.3	<0.001
At 3^rd^ week of 2^nd^ vaccine dose	105.2 ± 114.7	236.4 ± 41.4	<0.001

One healthy volunteer among study participants developed mild COVID-19 infection after the second dose of vaccination. No HD patient developed COVID-19 infection till the third week of vaccination.

## Discussion

Responsiveness to immunization in patients with renal disease may be low due to the changes in the function of the immune system, making them at increased risk of COVID-19 infection [2-3,5,7]. Therefore, patients with kidney diseases should be considered for COVID-19 vaccination as a priority [11]. Studies on patients on HD to evaluate the efficacy of COVID-19 vaccines are limited [12-13], and all studies have used either mRNA or virus vector-based vaccines. In order to understand the effectiveness of COVID-19 inactivated virus vaccines in patients on HD, we studied 81 participants, including HD patients and healthy individuals in our center after the COVID-19 vaccination, and compared the immune response between them.

Our study found a similar humoral response after COVID-19 vaccination in HD patients compared with healthy individuals with 80.6% seroconversion in healthy individuals and 72% in HD patients after the first dose and 93.5% in healthy individuals in HD patients at the third week of the second dose of vaccine.

Several studies have recently reported high immunogenicity of mRNA-based anti-SARS-CoV-2 vaccines in dialysis patients ranging from 81% to 96% [12,15-17]. Our results are in line with those published recently in this setting. Yanay et al. investigated dialysis patients and a control group (who had completed two doses of vaccination with the mRNA BNT162b2 vaccine) for anti-spike protein antibody response and observed them for up to 10 weeks found a lower response rate to the vaccine and a lower anti-spike antibody level in the dialysis group [16]. Lensy et al. from Germany assessed immunogenicity to a first mRNA- or vector-based SARS-CoV-2-vaccination dose in dialysis patients [13]. They found that two weeks after their first mRNA- or vector-based SARS-CoV-2 vaccination, hemodialysis patients demonstrated lower antibody-related response than peritoneal dialysis patients and healthy staff regardless of the type of vaccine. A lower humoral response was also reported in dialysis patients compared to a control group by Simon et al. [17]. The comparable response between healthy individuals and HD patients in our study may be attributed to the inactivated virus vaccine. However, further studies with a direct comparison between inactivated virus vaccine and mRNA- or vector-based SARS-CoV-2-vaccination will require to confirm these findings.

In our study, the age of the patients did not have any significant effect on antibody titer, contrary to some recent studies that showed an inverse correlation between age and antibody levels following the second dose of vaccination [12,16,18]. Lacson et al. observed that non-responders after two doses of BNT162b2 or mRNA-1273 were more likely to be female [19]; however, our study found no difference in response of IgG antibody titer at different times according to gender. 

Our study found that participants with a history of previous COVID-19 infection showed a better immunogenic response to vaccination. This confirms previous findings in patients on maintenance dialysis who recovered from COVID-19 [13,20]. Diabetes mellitus is a common comorbid condition that accounts for the high-risk factor in COVID-19 disease severity and mortality [21]. We detected that diabetic HD patients had a low antibody titer compared to non-diabetic. No such association has been studied in other research work so far and thus needs special attention to tailor the effective vaccination scheme for diabetic patients. Our study did not show a statistically significant impact on seroconversion rate and antibody titer with the type of vaccine (BBIBP-CorV or ConoVac), and HTN.

In our study, those vaccinated were not found to have COVID-19 infection during the study time period except for one healthy volunteer who developed a mild infection after the second dose. COVID-19 occurring in the normal population shortly after first vaccination has been described, and recently, reduced antibody response after the first dose of mRNA-based COVID-19 vaccine in hemodialysis patients was briefly reported [22]. Further studies need to be done to know if vaccination is effective for the prevention of different strains of COVID-19 infection. It will also be pertinent to study how long these antibodies remain in the blood.

This study was done at a single center with a small number of patients, and thus the generalizability of our study results is limited. In addition, only patients who received inactivated virus vaccines were studied, and only humoral (antibodies) immune response was tested.

## Conclusions

We observed no difference in early humoral response between healthy individuals and patients on HD after COVID-19 inactivated virus vaccination. The titer of IgG antibody increases corresponding to the doses of vaccine. Diabetic patients on HD showed a poorer immunogenic response to the vaccine than non-diabetic HD patients. Participants with a history of previous COVID-19 infection showed a better immunogenic response to vaccination. COVID-19 vaccination was found to be protective against COVID-19 infection in HD patients at least in the first three weeks after vaccination.
